# Use and usability of the dr. Bart app and its relation with health care utilisation and clinical outcomes in people with knee and/or hip osteoarthritis

**DOI:** 10.1186/s12913-021-06440-1

**Published:** 2021-05-10

**Authors:** Tim Pelle, Job van der Palen, Frank de Graaf, Frank H. J. van den Hoogen, Karen Bevers, Cornelia H. M. van den Ende

**Affiliations:** 1 Department of Rheumatology, Sint Maartenskliniek, Nijmegen, the Netherlands; 2 Department of Rheumatic Diseases, Radboud University Medical Center, PO Box 9011, 6500 GM Nijmegen, the Netherlands; 3 Department of Research Methodology, Measurement, and Data-Analysis, Behavioural, Management and Social Sciences, University of Twente, Enschede, The Netherlands; 4 Medical School Twente, Medisch Spectrum Twente, Enschede, the Netherlands; 5 Orikami B.V, Nijmegen, the Netherlands

**Keywords:** mHealth, Machine learning, Osteoarthritis, eHealth, Stand-alone, Application

## Abstract

**Background:**

Self-management is of paramount importance in the non-surgical treatment of knee/hip osteoarthritis (OA). Modern technologies offer the possibility of 24/7 self-management support. We developed an e-self-management application (dr. Bart app) for people with knee/hip OA.

The aim of this study was to document the use and usability of the dr. Bart app and its relation with health care utilisation and clinical outcomes in people with knee/hip OA.

**Methods:**

For this study we used backend data for the first 26 weeks of use by the intervention group (*N* = 214) of an RCT examining the effectiveness of the dr. Bart app. A central element of the dr. Bart app is that it proposes a selection of 72 preformulated goals for health behaviours based on the ‘tiny habits method’ (e.g. after lunch I rise 12 times from my chair to train my leg muscles). The usability of the app was measured using the System Usability Scale questionnaire (SUS), on a scale of 0–100. To assess the association between the intensity of use of the app and health care utilisation (i.e., consultations in primary or secondary health care) and clinical outcomes (i.e., self-management behaviour, physical activity, health-related quality of life, illness perceptions, symptoms, pain, activities of daily living) we calculated Spearman rank correlation coefficients.

**Results:**

Of the 214 participants, 171 (80%) logged in at least once with 151 (71%) choosing at least one goal and 114 (53%) completing at least one goal during the 26 weeks. Of those who chose at least one goal, 56 participants (37%) continued to log in for up to 26 weeks, 12 (8%) continued to select new goals from the offered goals and 37 (25%) continued to complete goals. Preformulated goals in the themes of physical activity (e.g., performing an exercise from the exercises library in the app) and nutrition (e.g., ‘eat two pieces of fruit today’) were found to be most popular with users. The mean usability scores (standard deviation) at the three and six month follow-ups were 65.9 (16.9) and 64.5 (17.5), respectively. The vast majority of associations between the intensity of use of the dr. Bart app and target outcomes were weak at ρ < (−) 0.25.

**Conclusions:**

More than one-third of people with knee/hip OA who started using the app, continued to use it up to 26 weeks, though usability could be improved. Patients appear to have preferences for goals related to physical activity and nutrition, rather than for goals related to vitality and education. We found weak/no associations between the intensity of use of the dr. Bart app and health care utilisation and clinical outcomes.

**Trial registration:**

**(21 September 2017):** Dutch Trial Register (Trial Number NTR6693/NL6505)

**Supplementary Information:**

The online version contains supplementary material available at 10.1186/s12913-021-06440-1.

## Background

Osteoarthritis (OA) of the knee/hip is the most common form of disability of movement and is characterised by pain, stiffness, and a decline in daily functioning. The primary approaches for non-surgical treatment in early stages of knee/hip OA are lifestyle education, exercise therapy, weight management and pain medication [1–4]. As OA is a chronic disease, a cornerstone of its non-surgical treatment is self-management. Self-management interventions offer patients guidance in improving their skills to take better care of themselves and take an active role in their disease management [5, 6], including skills navigating the health care system (i.e., making optimal use of primary and secondary health care options).

Compared with usual care, traditional self-management interventions (e.g. face-to-face education) show small benefits in the self-management skills, pain, and function of people with knee/hip OA [7]. Mobile health applications have the potential to enhance self-management 24/7 (i.e., 24 h a day, 7 days a week). Their growing and emerging popularity of eHealth applications have resulted in a proliferation of applications in the health domain; however, the majority of mobile health applications have not proven their effectiveness in clinical trials [8–12].

Given the potential of these modern technologies, we developed a fully automated stand-alone mobile health application (the ‘dr. Bart app’) to enhance self-management in people with knee/hip OA. The content of the dr. Bart app is based on the Fogg model for behavioural change, augmented with reminders, rewards and self-monitoring to reinforce app engagement [13, 23]. In a randomized evaluation, we found that the dr. Bart app did not impact health care utilisation (HCU), but resulted in small positive effects on pain, symptoms and activities of daily living [14]. However, a fundamental issue in eHealth research is non-usage attrition; a proportion of participants do not use the intervention at all, or use it sparsely [15].

Although the issue regarding non-usage attrition is well-known, most eHealth studies do not provide information regarding use, despite the possible diminishing of the effects of the intervention by low exposure rates [16–18]. Studies on stand-alone eHealth tools in other chronic diseases (e.g., diabetes and chronic pulmonary disease) showed that after a month the applications were used by fewer than 50% of participants [19]. Two studies evaluating stand-alone eHealth interventions assisting patients in their preparation for the first consultation with an orthopaedic surgeon, which could be used either one or 2 weeks prior to the consultation, found relatively high user rates (i.e. 70%) in people with OA [20, 21]. So far, there is little insight into whether the actual usage of stand-alone eHealth applications in people with OA enhances self-management and its determinants. Furthermore, it is likely that there is a dose response relationship between the (intensity of) use of an eHealth intervention and the clinical outcomes [22]; however, there has been little insight into the association between the extent of the use of (different components of) an app and its effects on target outcomes. The aim of this explorative study was therefore to quantify the use, identify the patterns of use and assess the usability of the dr. Bart app over half a year. Furthermore, we explored the association between the intensity of use of the dr. Bart app and its relation with HCU and clinical outcomes in people with knee/hip OA over half a year. Finally, we aimed to gain insight into the demographic and clinical characteristics of various types of users.

## Methods

### Design and setting

The data in the present study were collected as part of a randomised controlled trial (RCT), evaluating the effectiveness of the dr. Bart app on health care use and clinical outcomes, which was conducted by the Sint Maartenskliniek Nijmegen, the Netherlands, from 24 January 2018 to 7 January 2019. The original study is registered in the Dutch Trial Register (trial number NTR6693/NL6505) (https://www.trialregister.nl/trial/6505) as of 21 September 2017. All participants provided digital informed consent for participation. The ethical approval for this study was waived by the Medical Research Ethics Committee of the Radboud University Medical Centre, Nijmegen, the Netherlands (CMO Arnhem-Nijmegen, protocol number: 2017–3625) because the study was considered outside the remit of the law (Medical Research Involving Human Subjects Act). This study is reported according to the CONSORT guidelines.

### Participants and procedure

Participants were recruited through advertisements in local newspapers (i.e., the region of Nijmegen, the Netherlands), and throughout the Netherlands via campaigns on social media (i.e., Facebook, Twitter, and LinkedIn). Participants willing to participate were invited to the website (https://www.drbart.eu/) to check their eligibility. The inclusion criteria were: 1) having self-reported OA of the knee and/or hip (i.e., having a painful knee and/or hip, knee and/or hip pain > 15 days of the past month, morning stiffness < 30 min (knee) and/or < 60 min (hip)); 2) ≥ 50 years; 3) having an e-mail address; 4) possession of a smartphone or tablet and willing to download the dr. Bart application on one or more devices; and 5) able to read, write and sufficiently communicate in Dutch.

Exclusion criteria were as follows: 1) being wheelchair-bound; 2) diagnosis of (other) inflammatory rheumatic disease; 3) knee and/or hip replacements; and 4) scheduled for knee and/or hip joint arthroplasty in the next 6 months. Eligible participants were asked to provide their e-mail address and subsequently received a baseline assessment via CastorEDC, an electronic software application for data collection and management (https://www.castoredc.com/). Baseline and follow-up data at three and 6 months were taken from the intervention group (*N* = 214) of an RCT examining the effectiveness of the dr. Bart app, together with backend data (i.e., the technology component responsible for processing and storing the data) of the app over 26 weeks and used for the present analysis [13].

### Intervention

The dr. Bart app is a stand-alone mobile health application that was designed to enhance self-management and actively involve people with knee/hip OA in the management of their disease. This mobile health application is based on the Fogg model for behavioural change [23], augmented with other motivation-enhancing techniques such as reminders, rewards and self-monitoring, to reinforce app engagement and health behaviour. Users receive a daily push notification from dr. Bart. Additionally, the app automatically sends a push notification stating: “We have not seen you in a while. Do you think of your goals?” when a user has not opened the app for more than 7 days. The Fogg model, also known as the ‘tiny habits method’, utilises the concept of accumulating small goals to structurally change health behavior, and in the long run health outcomes. Machine learning techniques are used to propose tailored goals based on data collected in a personal profile and on previously selected and discarded goals. For the dr. Bart app, the machine learning comprised a dynamic model (contextual multi-armed bandit approach), proposing goals that are challenging, achievable and tailored for that specific user. The content and functionalities of the dr. Bart app were frozen during the study period (version 1.3.7), although bug fixes (e.g., failure to log in) and system failures were resolved. Further details on the theoretical framework, development and functionalities of the dr. Bart app have been published elsewhere [13]. Screenshots of the dr. Bart app are presented in Additional file 1: Appendix 1.

### Assessments

At the baseline, and three and 6 months after inclusion, participants received online questionnaires via CastorEDC (https://www.castoredc.com).

### Use of the dr. Bart app

Prior to start of the study, we decided which parameters of use should be logged and extracted from the backend of the app to quantitatively measure its use. These data were collected for the 26-week study period. The parameters of use were automatically logged and extracted for each participant. ‘Non-users’ were those participants who never logged in. To elaborate on the nature and extent of use of the app, we further classified use of the app as:

1. active with logins, but no further activity
2. active with choosing goals, but without completing goals
3. active with completing ≥ 1 goals

Users can choose more than one of the proposed goals simultaneously and goals can be completed more than once by the same user. The following indicators of use were extracted from the backend of the app: number of logins, number of unique chosen goals, number of unique goals completed, and total number of completed goals. Moreover, we quantified the use of information as the number of paragraphs read of the educational library (range 0–108), which indicates exposure to information.

For participants who chose at least one goal, we constructed Kaplan-Meier curves to illustrate the percentage of persons who used the app over time, based on the aforementioned indicators of use.

### Usability

We assessed the usability of the dr. Bart app with the System Usability Scale (SUS) at three and six months [24, 25]. The SUS is a 10-item questionnaire scored on a five-point Likert scale (“Strongly agree” to “Strongly disagree”. We calculated a total score ranging from 0 to 100, with a higher score indicating better usability. Additionally, we provided a free-text option after each question, so participants could elaborate on their given answers.

### Demographic and clinical characteristics

Demographic data were collected at the baseline. We assessed pain, symptoms, activities of daily living, quality of life, and physical functioning in sports and recreation, with subscales of either the Knee injury or Hip disability Osteoarthritis Outcome Score (KOOS or HOOS), ranging from 0 to 100, where a higher score indicates fewer problems in that domain [26, 27]. We assessed health-related quality of life using the EQ-5D-3L (0–1; with a higher score reflecting better health) [28]. Physical activity was assessed with the Short Questionnaire to Assess Health-Enhancing Physical Activity (SQUASH) [29]. Knowledge, skills and confidence to cope with one’s health were assessed with the Patient Activation Measure (PAM-13) questionnaire [30, 31]. We used the Illness Perception Questionnaire (IPQ) to assess the patient’s cognitive and emotional perception regarding their OA (0–80; higher score indicating more concerning views of OA) [32]. Moreover, we assessed both positive and negative treatment beliefs regarding various treatment modalities (i.e., physical activities, pain medication, physical therapy, injections, and joint replacement surgery) in knee and hip OA with the Treatment Beliefs in Osteoarthritis (TOA) questionnaire [33]. Psychometric properties are satisfactory to good. However, minimal clinically important difference is not yet available. We calculated mean sub scale scores ranging from 1 to 5 for the TOA.

### Statistical analysis

#### Data analysis

All statistical analyses were performed using Stata 13.1 [34]. The (missing) data were handled according to the recommendations of the specific questionnaire. For the PAM, we also calculated a total score when a maximum of two items of the questionnaire were missing, though the PAM recommends to only calculate a total score if no single item is missing. For the SUS questionnaire, we did not calculate a total score when two or more items were missing. Descriptive statistics were used to describe participant characteristics and parameters of use. In all analyses, we considered *p* < 0.05 to be statistically significant. Since this is an explorative study, we refrained from correcting for multiple testing.

#### Subgroup characteristics

In order to determine whether baseline characteristics could be used to predict the use of the app by subgroups of participants, parameters of use were taken as the dependent variable in univariate regression analyses, with the baseline characteristic as the independent variable.

#### Association between use and clinical outcomes

To assess the association between the intensity of (different indicators of) use of the app and changes in HCU and clinical outcomes over 6 months of follow-up, we calculated Spearman rank correlation coefficients. Additionally, we classified users into six groups for the four separate indicators of use (i.e., number of logins, number of chosen goals, number of completed goals and number of read paragraphs) based on backend data; non-users and a population split into five equal groups (i.e., quintiles). Subsequently, boxplots of relative differences in clinical outcomes were created for those six groups for the four different indicators of use.

## Results

The original randomised controlled trial included 427 participants with knee/hip OA [14]. Of those, 214 participants were randomised into the intervention group and subsequently included in the current explorative study. The mean age of these participants was 62.1 years (SD 7.7), with the majority being female (68.7%). Most participants had symptoms predominantly in their knee(s) (73.4%), and 60 % had OA symptoms for less than 5 years (Table 1).

**Table 1:** Baseline and clinical characteristics of the participants in the study

	Dr. Bart app group (*N* = 214)
Age, years; mean (SD)	62.1 (7.7)
Female, *n* (%)	147 (68.7)
Body mass index, kg/m^2^; mean (SD)	27.8 (5.1)
Level of education (≤ 12 years), *n* (%)	56 (28.0)
Main OA location	
- Knee, *n* (%)	157 (73.4)
Duration of symptoms, *n* (%)	
- < 1 year	25 (11.7)
- 1–5 years	104 (48.6)
- 6–10 years	49 (22.9)
- > 10 years	36 (16.8)
*Clinical characteristics*	
Symptoms^a^ (0–100); mean (SD)	57.7 (16.3)
Pain^a^ (0–100); mean (SD)	57.5 (15.5)
Activities of daily living^a^ (0–100); mean (SD)	58.5 (19.7)
Activities^a^ (0–100); mean (SD)	32.6 (23.9)
Quality of life^a^ (0–100); mean (SD)	38.0 (17.5)
Self-management behaviour^b^ (13–52); mean (SD)	*40.8 (5.3)*
Physical activity, total hours/week; mean (SD)	*31.6 (21.2)*
Health-related quality of life (0–1); mean (SD)	*0.72 (0.19)*
Health-related quality of life (slider) (0–100); mean (SD)	*70.9 (15.5)*
Illness Perceptions (range 0–80); mean (SD)	*43.1 (8.9)*

**Abbreviations:** *SD* Standard Deviation; *n* number; *OA* osteoarthritis; *PA* physical activity. ^a^Measured with either KOOS or HOOS. ^b^Measured with the Patient Activation Measure Short Form (PAM-13)

### Use of the dr. Bart app

Among the participants, 171 (79.9%) were active with logins, 151 (70.6%) were active with choosing goals and 113 (52.8%) were active with completing goals. The remaining 20.1% of participants did not log in to the app over the course of the study. We did not find relevant differences in the baseline characteristics of those who were active with the dr. Bart app and those who were less active or inactive (Additional file 1: Appendix 2).

In total, participants logged in 7006 times, chose 1062 goals, completed 884 unique goals and completed 9229 goals over the 26 weeks (Table 2). The median number of paragraphs read of the educational library was 42 (interquartile range (IQR): 18–84) for participants who were active with completing goals (Table 2), while 50% of participants who were active and chose goals read more than 30 paragraphs in the educational library. Paragraphs about OA and its complaints, progression, conservative treatment options, as well as the exercise library, were read more often than paragraphs related to the pathogenesis of OA, pharmacological care, assistive technologies and surgical treatments (Additional file 1: Appendix 3).

**Table 2:** Indicators of use over 26 weeks, presented for three pre-specified groups of users based on their usage activity (*N* = 171). Of note, non-users are not included in this Table

	Logged in, but no further activity (*n* = 20)	Chose ≥1 goal, but did not complete goals (*n* = 38)	Completed ≥1 goal (*n* = 113)
Number of logins, median [IQR]	2.5 [1.5; 4.5]	4 [3; 11]	33 [16; 89]
Length of use, mean days median days [IQR]	26.1 4.5 [1; 29.5]	66.0 60.5 [6;102]	115.9 144 [63; 173]
SUS score at 3 month follow-up, mean (SD)	65.2 (17.7) (*N* = 7)	55.0 (15.1) (*N* = 14)	68.6 (16.5) (*N* = 72)
SUS score at 6 month follow-up, mean (SD)	51.3 (15.5) (*N* = 9)	52.0 (16.2) (*N* = 10)	69.2 (16.9) (*N* = 63)
Number of paragraphs read (range 0–108), median [IQR]	0 [0; 11.5]	5.5 [1; 21]	42 [18; 84]
Number of unique chosen goals, median [IQR]	N/A	2 [1; 3]	6 [3; 11]
Number of unique completed goals, median [IQR]	N/A	N/A	5 [3; 10]
Number of total completed goals median [IQR]	N/A	N/A	35 [11; 117]

**Abbreviations:** *IQR* interquartile range; *SD* Standard Deviation; *n* number; *OA* osteoarthritis

**Fig. 1:**
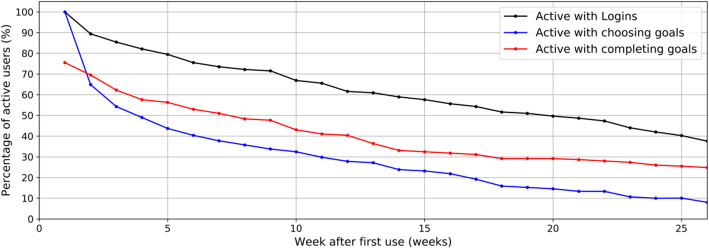
Percentage of users that are active with logins, choosing goals or completing goals over time as a fraction of the users that chose at least one goal during the study (*N* = 151)

Fig. 1 shows the proportion of participants actively using the dr. Bart app over half a year, with separate lines for the three indicators of use. Of the participants who chose at least one goal, 38% were active with logins after 26 weeks, 8% were active with choosing goals and 25% were active with completing goals. Appendices 4 and 5 show the median and IQR of the number of cumulative logins and completed goals over time of users who chose at least one goal, respectively.

Goals related to physical activity were relatively most often chosen (times chosen divided by times proposed) and completed (times completed divided by times chosen), and goals related to nutrition were more popular than goals related to vitality or education. Appendices 6 and 7 show the five relatively most and least often chosen and completed goals.

### Usability

After three and three months of follow-up, the mean usability scores were 65.9 (SD 16.9) and 64.5 (SD 17.5), respectively. People who more intensively used the app, gave higher usability scores (Table 2). Participants rated the main features (i.e. education and exercise library) of the app positively in the additional free-text option of the SUS (Additional file 1: Appendix 8). Moreover, the ability to access this information at any given time and place was considered important, as well as to incorporate the ‘tiny habits’ easily in daily life. On the other hand, 14 participants stated that the app did not provide any new information or exercises or that they did not always carry their mobile phone. Moreover, 13 participants did not see the benefits of using such an application (Additional file 1: Appendix 8).

### Subgroup characteristics

We could not (clearly) distinguish between non-users and different intensities of users, based on age, gender, body mass index (BMI), living situation, level of education, or main OA location (Additional file 1: Appendix 9).

### Association between use and clinical outcomes

We found Spearman’s rank correlation coefficients ranging from − 0.20 to 0.30 between different indicators of use and HCU and clinical outcomes (Table 3). We found a statistically significant correlation between visiting a general practitioner (yes/no) and the number of unique chosen goals (*ρ* = 0.25) and unique goals completed (*ρ* = 0.28).

**Table 3:** Spearman rank correlation coefficients between different indicators of use and health care utilisation and clinical outcomes (relative difference between baseline and 6 month follow-up)

	Logins	Unique goals chosen	Unique goals completed	Total goals completed	Paragraphs read
Visited a secondary health care provider? (yes/no)	0.02	0.05	0.08	0.06	0.09
Visited a general practitioner? (yes/no)	0.07	**0.25 (*P*** **= .0139)**	**0.28 (*P*** **= .0106)**	0.09	0.03
Pain^a^	−0.17	−0.18	− 0.22	−0.16	− 0.07
Symptoms^a^	−0.07	− 0.11	−0.15	− 0.21	−0.19
Activities of daily living^a^	0.08	−0.03	−0.09	0.06	0.02
Quality of life^a^	−0.01	−0.07	− 0.11	−0.01	− 0.07
Activities^a^	0.09	−0.19	0.01	0.22	−0.08
Health-related quality of life *(EQ-5D-3L)*	−0.02	−0.07	− 0.12	−0.06	− 0.131
PA, total hours	−0.03	0.11	0.05	−0.00	0.081
Patient Activation Measure	0.05	0.02	−0.04	0.08	−0.11
Illness Perceptions	0.04	0.07	0.02	0.04	0.06

^a^Measured using either KOOS or HOOS

Negative beliefs about physical activity, medication and physical therapy were associated with lower app use (Additional file 1: Appendix 10).

A visual inspection of boxplots showed an absence of a dose-response relationship (data not shown).

## Discussion

The aim of this study was to document the use and usability of the dr. Bart app and to examine intensity of use of the app and its relation with HCU and clinical outcomes. The results of this study show that more than one-third of participants who were offered the dr. Bart app persistently used it over their first 6 months of access. The two main features of the app, goal setting and the educational library (including the exercise library), were extensively used. More than half of the participants completed at least one goal, with goals related to physical activity and nutrition being the most popular. In the educational library, participants were predominantly interested in general information regarding OA, complaints (specifically fatigue), treatment options, prognosis, and the exercise library. After half a year, a quarter of users still used the app to set and complete goals and two-fifth of users still opened the app. We could not identify differences in the characteristics of non-users and users of the dr. Bart app. Moreover, we were not able to demonstrate a dose-response relationship between different indicators of use and HCU or clinical outcomes.

To our knowledge little is known about the use of stand-alone mobile health applications in OA. Our user rates are in line with two studies evaluating stand-alone mobile health applications assisting people with OA in their preparation for their first consult with an orthopaedic surgeon [20, 21]; however, these applications had relatively short time frames (i.e., one or 2 weeks prior to consultation) and a different focus, which might have resulted in higher user rates in these studies. Stand-alone applications in other chronic diseases (chronic pulmonary disease or diabetes) are not directly comparable, as the use of these applications is more likely to result in short-term benefits (e.g. fewer exacerbations and betters glucose levels), and are consequently more likely to be used than the dr. Bart app. Nevertheless, the user rates over time observed in our study were even higher than those observed in stand-alone eHealth applications for the self-management of other chronic diseases [35–38]. Additionally, the use of the dr. Bart application is comparable with the use of a self-management application embedded in clinical practice (blended intervention in people with OA) [39], despite the use of blended interventions being more likely to result in higher user rates than stand-alone applications [16]. Taken together, we conclude that the usage rate of the stand-alone dr. Bart application is relatively high. This could be explained by the application of different reinforcement techniques (i.e. rewards, reminders and self-monitoring) to reinforce engagement with the dr. Bart app [13].

The majority of users consulted the educational library on a regular basis and were particularly interested in general information regarding OA, complaints, progression and, (non-pharmacological) conservative treatment options. Interestingly, information about the services provided by health care professionals was less frequently consulted, despite the navigation of the health care system being an important aspect of self-management. A possible explanation is that the majority of our sample is highly educated, which is known to be associated with possessing better skills of navigating the health care system [40]. Also, information with respect to the pathogenesis of OA, pharmacological care, walking aids, assistive technology, and surgical treatments were found to be less popular. This is an interesting finding, as these treatments are also considered important in the treatment of OA; however, topics considered important in the guidelines, do not necessarily reflect the educational needs of patients [41]. Our study provides insights into the educational needs of patients with knee/hip OA and offers starting points for optimising patient education.

Contrary to expectations, we did not find associations between the (intensity of) use of the dr. Bart app and HCU and clinical outcomes. Studies on web-based interventions in patients with mental disorders suggest that certain levels of use result in therapy saturation, and that patients are most likely to obtain benefits from the intervention early on [22]. The lack of any strong relationship between use and HCU and clinical outcomes might be explained by this model; users might have reached a plateau in which they did not benefit from additional use, with most benefits of the intervention coming early on. Given the nature of the data collected (we only have clinical outcomes after three and six months of follow-up), we were not able to assess the relationship between the early use of the app and its relationship with HCU and clinical outcomes. Future studies should assess the exact relationship between usage and HCU and clinical outcomes.

The usability of the app (assessed with the SUS) could be improved, as it does not reach an acceptable score of 70, which in turn might result in higher usage rates. However, a study with a satisfactory to good usability score regarding an application for post-operative self-reporting after colorectal surgery showed that participants did not use the app or used it only once [42]; thus, high usability alone is not sufficient to motivate patients to use eHealth applications. This is in line with the Technology Acceptance Model, which states that actual system use is not only dependent of the perceived ease-of-use (i.e., usability), but also on the perceived usefulness. Perceived usefulness is described as “the degree to which a person believes that using a particular technology would enhance his/her performance”. It is therefore important that participants see the necessity and benefits of using an application, in addition to the usability of the technology itself. Qualitative information about the usability of the app, not covered by the SUS, could help to identify targets to improve the usability [43]. For future studies, we recommend performing qualitative studies in parallel with the evaluation of a new intervention as this is essential to derive useful insights from end-users. Moreover, qualitative studies allow the elicitation of suggestions for improvement, which can be incorporated in new versions as well as the identification of facilitators and barriers for the use of the intervention.

### Strength and limitations

The current study has several limitations that should be taken into account when interpreting the results. First, sample selection bias is likely to be apparent, as participants were willing to participate in research on a mobile health application, which in turn might induce the Hawthorne effect (i.e., a change in behaviour of participants due to their awareness of being observed). Moreover, it is unknown why 20% of participants did not open the app. It is therefore conceivable that user rates are lower and non-use is higher when the application is applied in clinical practice. Furthermore, the results of this exploratory study were based on four different indicators of use, since a consensus on a definition of use and how to measure it is lacking. Nevertheless, we think that applying four different indicators of use, resulted in a thorough understanding of the use of the app. Since this is an explorative study, we chose not to formulate particular hypotheses in advance, which should be taken into account when interpreting the results. As said, the current study was exploratory in nature and we did not define the intended usage prior to the start of the study. Since intended use of a technology forms an important element of the definition of adherence. In future investigations, it might be useful to operationalise the intended use of eHealth technologies based on theory, evidence or rationale as the intended use of a technology forms an important element of the definition of adherence.

## Conclusions

This study is the first to elucidate the use and usability of a stand-alone mobile health application in people with knee/hip OA. A considerable proportion of participants persistently used the dr. Bart app for up to half a year, confirming that a stand-alone mobile health application has the potential to reach the target population. However, we were not able to demonstrate a dose-response relationship between use of the app and HCU and clinical outcomes. Additionally, this study provides insights into the educational needs of patients with knee/hip OA. In conclusion, we think the dr. Bart app has the potential to serve as a trustworthy tool to provide education and facilitate goal setting in people with knee/hip OA.

## Supplementary Information

**Additional file 1: Appendix 1.** Screenshots of the dr. Bart app. **Appendix 2.** Baseline characteristics of participants in the study per indicator of use and non-users. **Appendix 3.** The 5 most and least often read paragraphs. **Appendix 4.** Median and interquartile range of the number of cumulative completed goals over time of the active users (*n* = 151). **Appendix 5.** Median and interquartile range of the number of cumulative logins over time of the active users (*n* = 151). **Appendix 6.** The 5 relatively most and least often completed goals (i.e., times completed / times chosen). **Appendix 7.** The 5 relatively most and least often chosen goals (i.e., times chosen / times proposed). **Appendix 8.** Some responses to the free-text option of the SUS. **Appendix 9.** Regression coefficient and 95% confidence interval of the relation between baseline characteristics and different parameters of use. **Appendix 10.** Spearman rank correlation coefficients between different indicators of use and beliefs regarding 5 treatment modalities in knee/hip OA as measured with the treatment beliefs in osteoarthritis questionnaire (TOA) (relative difference between baseline and six month follow-up).

## Data Availability

The datasets used and/or analysed in the present study are available from the corresponding author upon reasonable request.

## Abbreviations

OA: Osteoarthritis
SUS: System Usability Scale
app: application
RCT: Randomized Controlled Trial
SD: Standard Deviation
KOOS or HOOS: Knee injury/Hip disability Osteoarthritis Outcome Score
PA: Physical Acitivity
PAM: Patient Activitation Measure
IPQ: Illness Perception Questionnaire
HCU: health care utilization
IQR: Interquartile Range
BMI: Body Mass Index
TAM: Technology Acceptance Model
