# Supplementary material for: Use and usability of the dr. Bart app and its relation with health care utilisation and clinical outcomes in people with knee and/or hip osteoarthritis
Source: BMC Health Serv Res. 2021 May 10;21:444. doi: 10.1186/s12913-021-06440-1 (PMC8112040; doi:10.1186/s12913-021-06440-1)
Supplement: Supplementary file 1 — Additional file 1: Appendix 1. Screenshots of the dr. Bart app. Appendix 2. Baseline characteristics of participants in the study per indicator of use and non-users. Appendix 3. The 5 most and least often read paragraphs. Appendix 4. Median and interquartile range of the number of cumulative completed goals over time of the active users (n = 151). Appendix 5. Median and interquartile range of the number of cumulative logins over time of the active users (n = 151). Appendix 6. The 5 relatively most and least often completed goals (i.e., times completed / times chosen). Appendix 7. The 5 relatively most and least often chosen goals (i.e., times chosen / times proposed). Appendix 8. Some responses to the free-text option of the SUS. Appendix 9. Regression coefficient and 95% confidence interval of the relation between baseline characteristics and different parameters of use. Appendix 10. Spearman rank correlation coefficients between different indicators of use and beliefs regarding 5 treatment modalities in knee/hip OA as measured with the treatment beliefs in osteoarthritis questionnaire (TOA) (relative difference between baseline and six month follow-up). [file 12913_2021_6440_MOESM1_ESM.docx]

**Appendix 1** Screenshots of the dr. Bart app


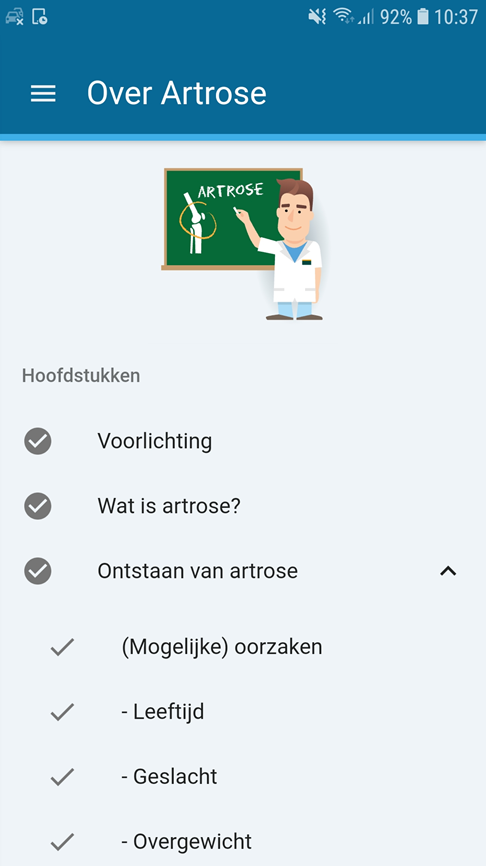

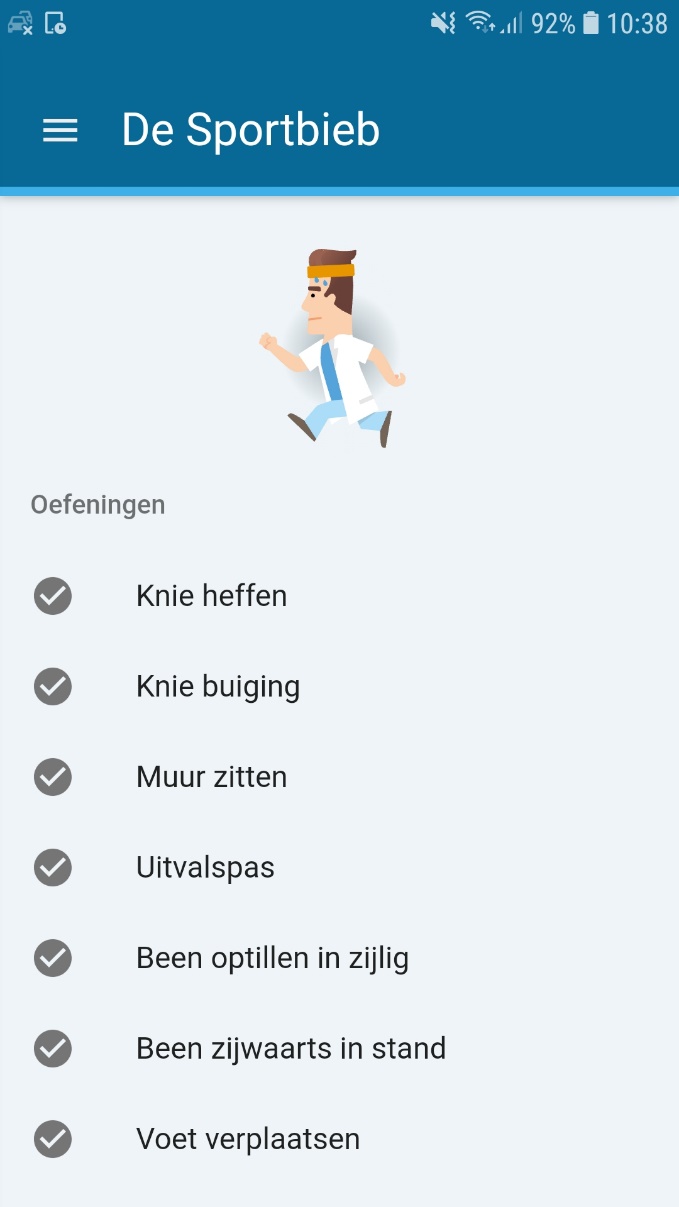

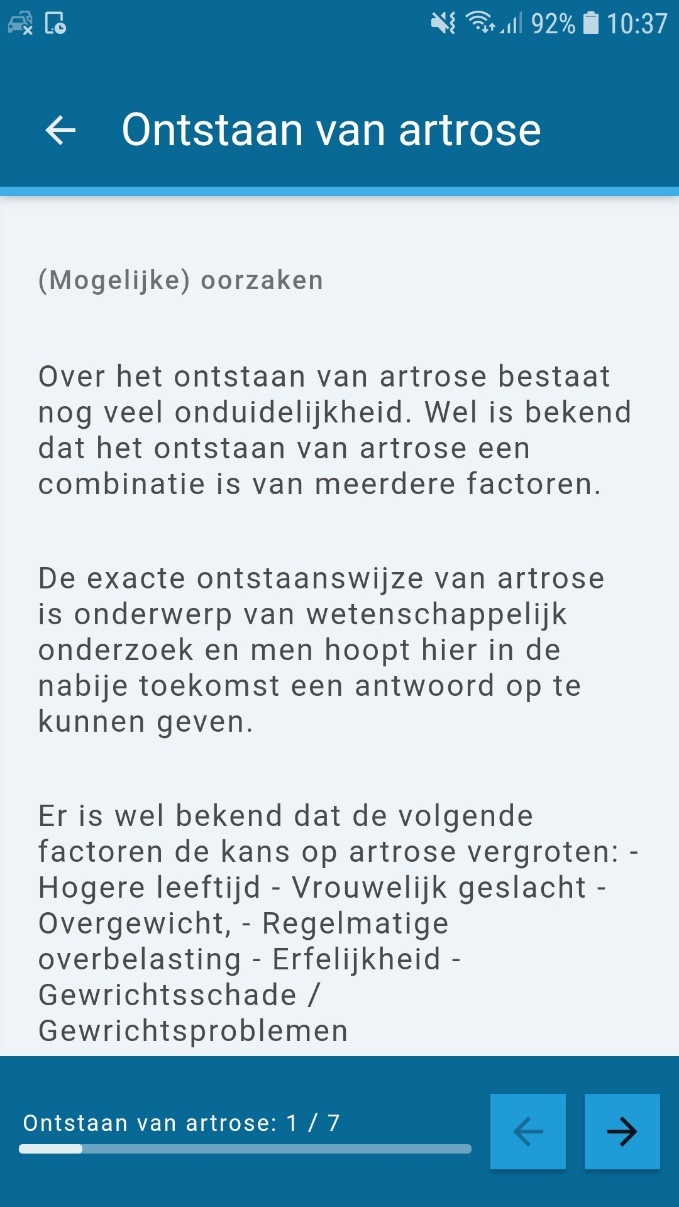


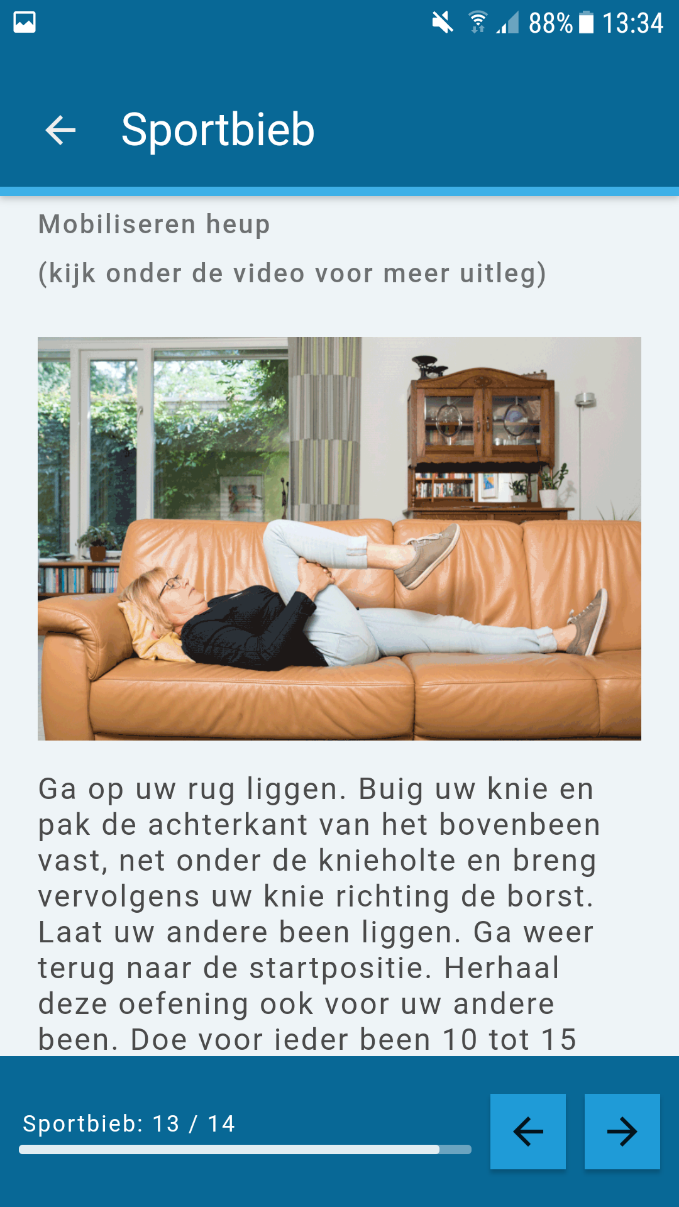

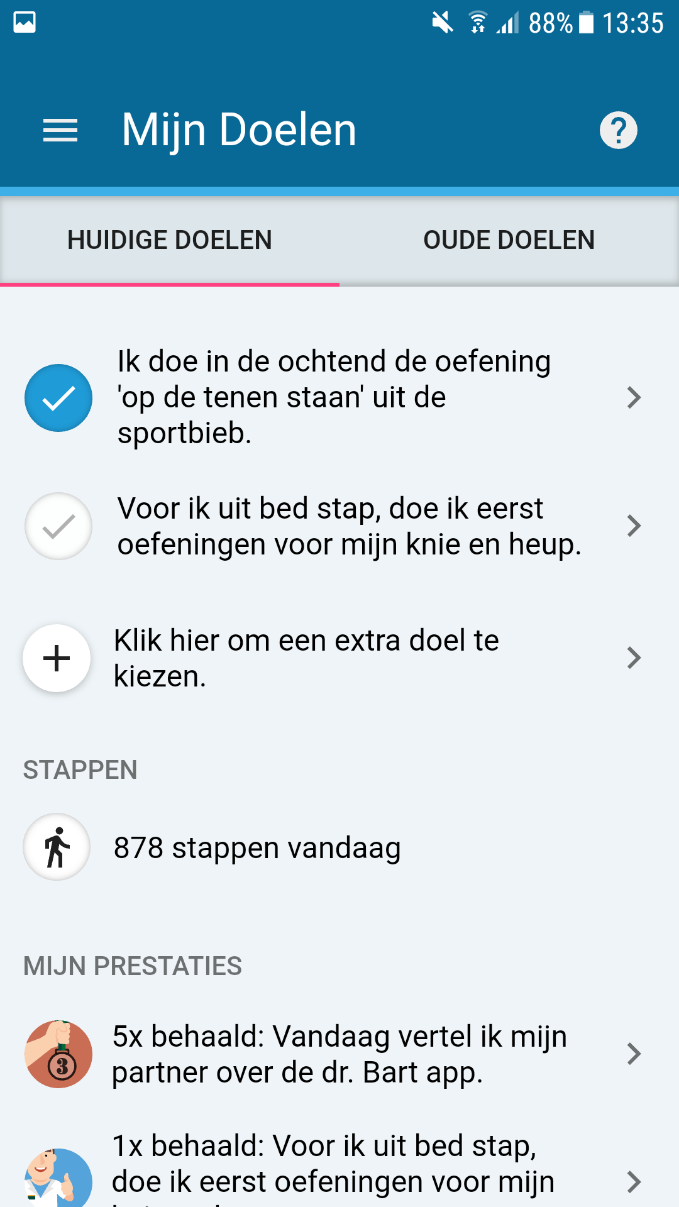

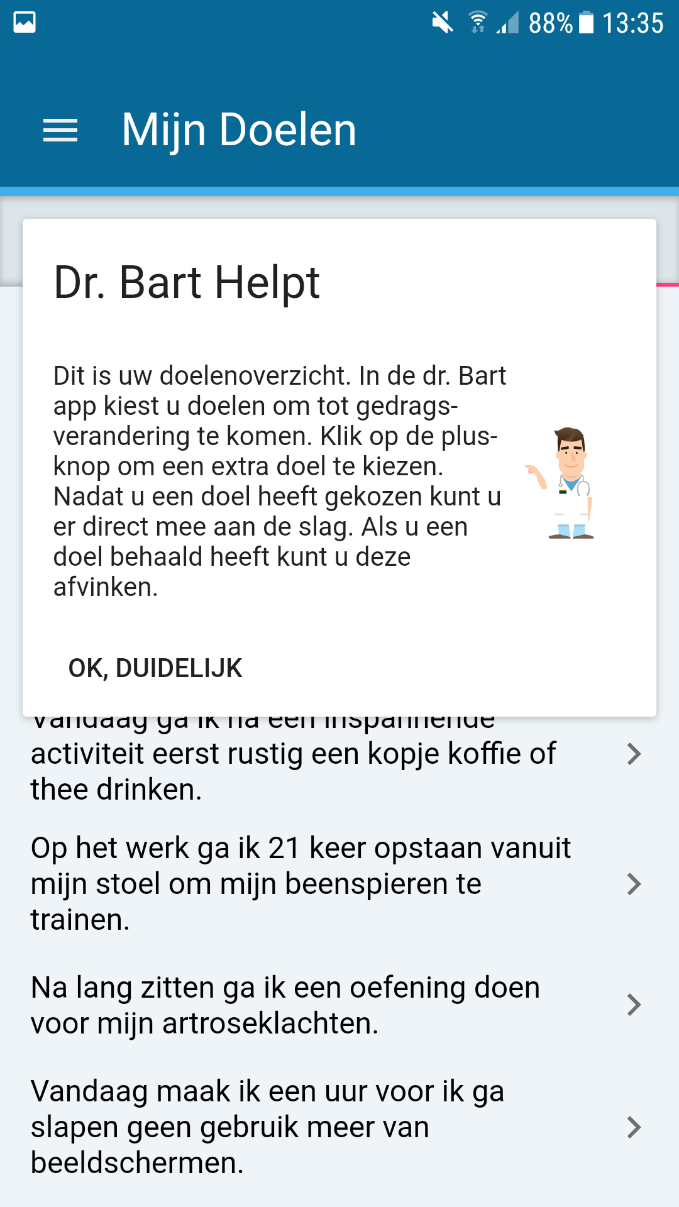


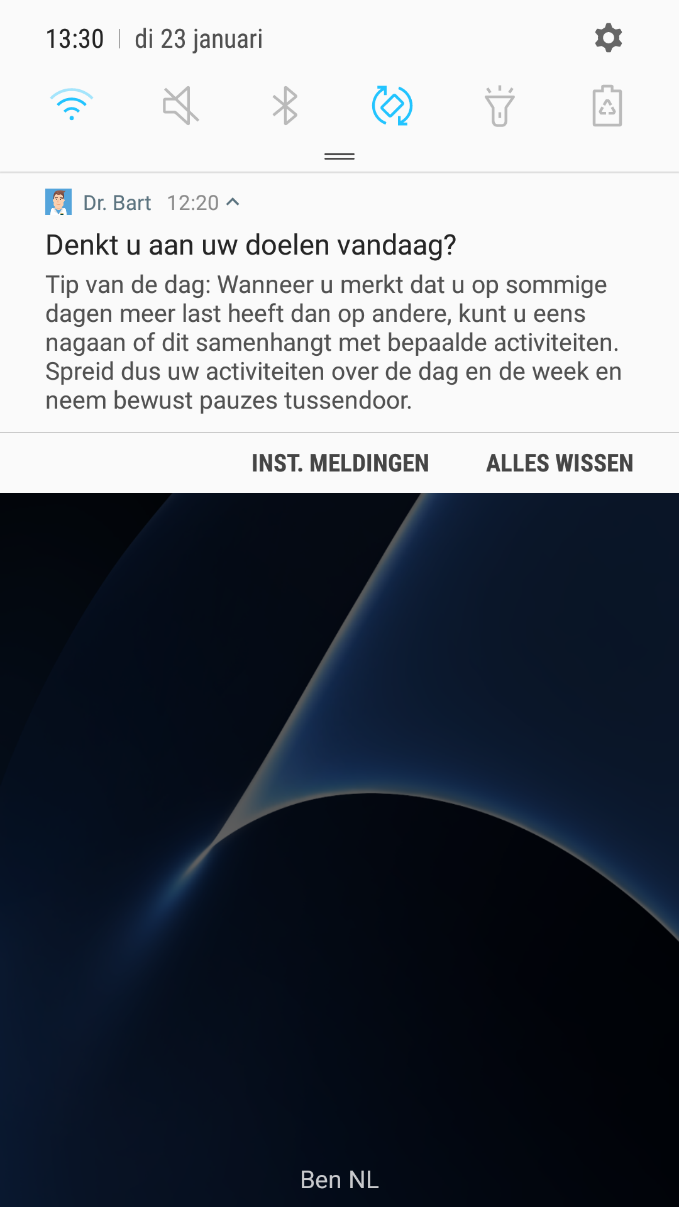


**Appendix 2** Baseline characteristics of participants in the study per indicator of use and non-users

|  | Non-user  (N=43) | Logged in, but no further activity  (N=20) | Chose ≥ 1 goal, but did not complete goals  (N=38) | Completed ≥ 1 goal  (N=113) |
| --- | --- | --- | --- | --- |
| Age, years; mean (SD) | 62.2 (7.1) | 63.9 (6.4) | 64.7 (10.5) | 60.8 (6.8) |
| Female, n(%) | 31 (72.1) | 12 (60.0) | 23 (60.5) | 81 (71.7) |
| Body Mass Index, kg/m^2^; mean (SD) | 29.0 (6.0) | 28.9 (5.6) | 27.5 (4.8) | 27.2 (4.7) |
| Level of education (≤ 12 years, n(%) | 13 (33.3) | 5 (29.4) | 12 (33.3) | 26 (24.1) |
| Main OA location |  |  |  |  |
| - Knee, n(%) | 31 (72.1) | 16 (80.0) | 28 (73.7) | 82 (72.6) |
| Duration of symptoms, n(%) |  |  |  |  |
| - < 1 year | 5 (11.6) | 2 (10.0) | 7 (18.4) | 11 (9.7) |
| - 1 – 5 years | 23 (53.5) | 9 (45.0) | 18 (47.4) | 54 (47.8) |
| - 5 – 10 years | 9 (20.9) | 2 (10.0) | 11 (29.0) | 27 (23.9) |
| - > 10 years | 6 (14.0) | 7 (35.0) | 2 (5.3) | 21 (18.6) |

**Abbreviations:** SD, Standard Deviation; n, number; OA, osteoarthritis.

**Appendix 3** The 5 most and least often read paragraphs

| **Most popular paragraphs** | **Least popular paragraphs (<20%)** |
| --- | --- |
| 1. General information regarding osteoarthritis. | 1. Information about the four themes in the dr. Bart app. |
| 1. What is osteoarthritis? | 2. If drugs do not help enough, what other treatment are available? |
| 1. Complaints (especially fatigue) | 3. With what frequency and intensity should I exercise? |
| 1. Prognosis of OA | 4. Are there any specific points I need to consider with regard to my job activities? |
| 1. Treatment of osteoarthritis | 5. How long does an artificial joint (joint replacement) last? |

**Appendix 4** Median and interquartile range of the number of cumulative completed goals over time of the active users (*n* = 151)


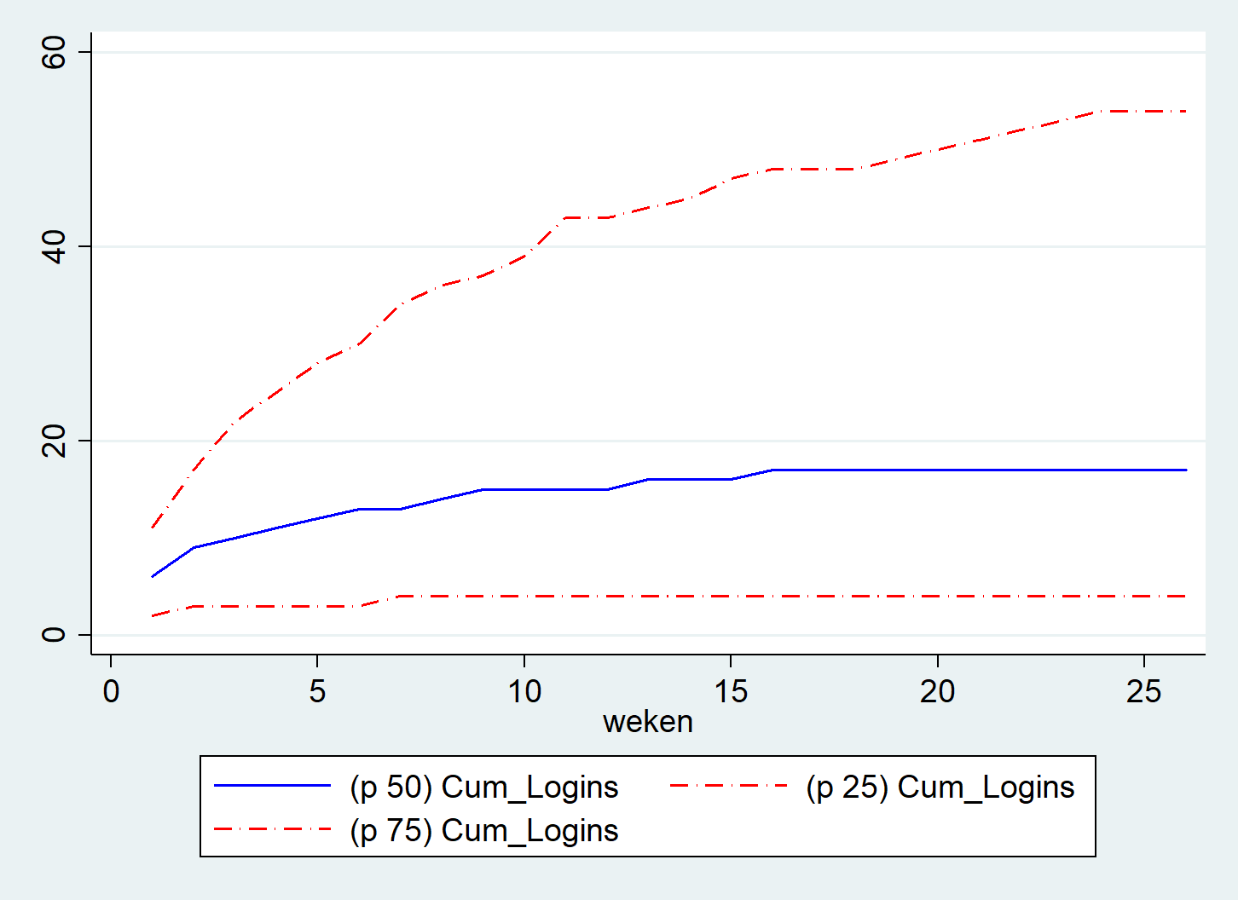


**Appendix 5** Median and interquartile range of the number of cumulative logins over time of the active users (*n* = 151)

**Appendix 6** The 5 relatively most and least often completed goals (i.e. times completed / times chosen) .

| **The 5 relatively most often completed goals.** | **The 5 relative least often completed goals** |
| --- | --- |
| Today I participate in Netherlands on the move {trigger] on the television. | Today I'm telling to {spouse/neighbor} what osteoarthritis comprises. |
| Today I walk [number of] steps. | Today I'm telling {spouse/neighbor} about the dr. Bart app. |
| Today I won't spoon more than once with dinner. | Today I read a chapter about osteoarthritis from the education library. |
| I'm going to perform [number of exercises] from the exercise library {trigger}. | I'm going to swim [number of laps] {trigger}. |
| {Trigger} I 'm going to get up [number of times] from my chair to train my lug muscles. | Today I put a sweetener in the coffee or tea instead of sugar. |

**Appendix 7** The 5 relatively most and least often chosen goals (I.e. times chosen / times proposed).

| **The 5 relatively most and least often chosen goals.** | **Least often chosen goals** |
| --- | --- |
| During {trigger} I drink a glass of water. | Today I participate in Netherlands on the move {trigger] on the television. |
| Today I won't spoon more than once during dinner. | Today I'm telling to {spouse/neighbor} what osteoarthritis comprises. |
| I'm going to improve the stability of my legs by doing an exercise from the exercise library during {trigger}. | Today I get out of the bus one stop earlier than usual and walk the rest. |
| Today I don't drink any sweetened drinks (such as soda or fruit juice). | Today I divide my household activities over the day. |
| During {trigger} I'm going to stand on my toes and slowly lower myself again and repeat this [number] times. | {Trigger} I'm going to walk for [number] minutes. |

**Appendix 8** Some responses to the free-text option of the SUS

*“I only keep using the exercise library”.*

*“Personally, the exercises are easy and easy to sustain”*

*“Besides the (exercise) library, I do not think the app has any added value”*

*“The exercises are beneficial. I cannot judge the other elements as beneficial”*

*“Use of the app encourage me to do exercises and learn about different behaviors (e.g. eating and drinking)”*

*“Lots of useful info in the library”*

*“Good structure and clear information”*

*“Lots of information that you can go through in your own pace”*

*“I used to be afraid of movement ….. Now I have more confidence due to the provided information”*

*“Info and instructions are clear”*

*“Do not see the benefit of the app compared to an information leaflet”*

*“The app does not add anything for me”*

*“The app does not have any added value for me”*

*“For the elderly (70+), using an app seems to difficult, but this applies to all electronical devices”*

*“Besides the (exercise) library, I do not think the app has any added value”*

*“Quickly bored of using the app”*

*“I do not think the app really adds any value once you have read the library and performed the exercises”.*

**Appendix 9** Regression coefficient and 95% confidence interval of the relation between baseline characteristics and different parameters of use

|  | Age | Gender^a^ | BMI | Main OA location^b^ | Level of education^c^ | Duration of symptoms^d^ |
| --- | --- | --- | --- | --- | --- | --- |
| Logins | 0.0  (-1.1; 1.1) | 9.9  (-8.3; 28.2) | -1.5  (-3.2; 0.3) | 8.9  (-10.5; 28.3) | -10.1  (-29.3; 9.1) | -8.8  (-35.3; 17.8) |
| Unique Goals Chosen | 0.0  (-0.1; 0.2) | 0.8  (-1.9;3.4) | -0.8  (-0.3; 0.2) | 0.4  (-3.1; 2.4) | -0.9  (-3.6; 1.9) | -1.5  (-5.3; 2.2) |
| Unique Goals Completed | 0.1  (-0.1; 0.3) | 0.1  (-2.9; 3.1) | -0.1  (-0.3; 0.2) | -0.5  (-3.6; 2.6) | -1.2 (-4.4; 1.9) | -2.9  (-7.5; 1.7) |
| Total goals completed | 2.7  (-0.1; 5.5) | 9.1  (-34.2; 52.4) | -3.2  (-7.3; 0.9) | 14.1  (-29.6; 57.8) | -34.7  (-81.5; 12.1) | -30.0  (-95.6; 35.6) |
| Paragraphs | -0.8  (-1.5; -0.2) | 10.2  (-0.9; 21.4) | -1.0  (-2.1; 0.1) | 3.2  (-8.7; 15.1) | 12.9  (0.8; 24.9) | 19.0  (3.0; 35.1) |

**^a^** Male as reference category
^b^ Knee as reference category
**^c^** Less than 12 years as reference category
**^d^** duration of symptoms > 10 years as reference category

**Appendix 10** Spearman rank correlation coefficients between different indicators of use and beliefs regarding 5 treatment modalities in knee/hip OA as measured with the treatment beliefs in osteoarthritis questionnaire (TOA) (relative difference between baseline and 6 month follow-up)

| *TOA (1-5)* | Logins | Unique Goals Chosen | Unique Goals Completed | Total goals completed | Paragraphs |
| --- | --- | --- | --- | --- | --- |
| PA Pos.  PA Neg. | -0.01  **-0.38* (*p* = .0002)** | -0.18  -0.18 | -0.22  -0.11 | -0.11  -0.22 | -0.17  -0.20 |
| Med. Pos.  Med. Neg. | 0.08  -0.19 | 0.05  **-0.30* (*p* = .0059)** | 0.14  **-0.31* (*p* = .0071)** | 0.19  **-0.27* (*p* = .0224)** | 0.11  **-0.24* (*p* = .0222)** |
| PT Pos.  PT Neg. | 0.01  -0.12 | 0.03  **-0.23* (*p* = .0463)** | 0.05  **-0.32* (*p* = .0001)** | -0.13  -0.09 | -0.09  0.01 |
| Inj. Pos.  Inj. Neg. | -0.01  0.06 | 0.09  -0.05 | 0.11  0.01 | -0.03  0.14 | 0.09  0.07 |
| TJR Pos.  TJR Neg. | 0.02  -0.04 | -0.01  -0.06 | 0.05  -0.17 | 0.02  -0.02 | -0.14  -0.05 |

**Abbreviations:** PA, physical activity; Med., medication; PT, physical therapy; Inj, injections; TJR, total joint arthroplasty; Pos., positive; Neg., Negative.
